# Effects of local hypothermia–rewarming on physiology, metabolism and inflammation of acutely injured human spinal cord

**DOI:** 10.1038/s41598-020-64944-y

**Published:** 2020-05-15

**Authors:** Mathew J. Gallagher, Florence R. A. Hogg, Siobhan Kearney, Marcel A. Kopp, Christian Blex, Leonarda Serdani, Oliver Sherwood, Jan M. Schwab, Argyro Zoumprouli, Marios C. Papadopoulos, Samira Saadoun

**Affiliations:** 1 Academic Neurosurgery Unit, Molecular and Clinical Sciences Institute, St. George’s, University of London, London, UK; 2 Clinical and Experimental Spinal Cord Injury Research (Neuroparaplegiology), Charité - Universitätsmedizin Berlin, Charitéplatz 1, 10117 Berlin, Germany; 3 Berlin Institute of Health, QUEST-Center for Transforming Biomedical Research, Berlin, Germany; 4 Belford Center for Spinal Cord Injury, Departments of Neurology, Neuroscience and Physical Medicine and Rehabilitation, The Neurological Institute, The Ohio State University, Wexner Medical Center, Columbus, OH 43210 USA; 5 Neuro-Anaesthesia and Neuro-Intensive Care Unit, St. George’s Hospital, London, UK

**Keywords:** Spinal cord diseases, Translational research

## Abstract

In five patients with acute, severe thoracic traumatic spinal cord injuries (TSCIs), American spinal injuries association Impairment Scale (AIS) grades A–C, we induced cord hypothermia (33 °C) then rewarming (37 °C). A pressure probe and a microdialysis catheter were placed intradurally at the injury site to monitor intraspinal pressure (ISP), spinal cord perfusion pressure (SCPP), tissue metabolism and inflammation. Cord hypothermia–rewarming, applied to awake patients, did not cause discomfort or neurological deterioration. Cooling did not affect cord physiology (ISP, SCPP), but markedly altered cord metabolism (increased glucose, lactate, lactate/pyruvate ratio (LPR), glutamate; decreased glycerol) and markedly reduced cord inflammation (reduced IL1β, IL8, MCP, MIP1α, MIP1β). Compared with pre-cooling baseline, rewarming was associated with significantly worse cord physiology (increased ICP, decreased SCPP), cord metabolism (increased lactate, LPR; decreased glucose, glycerol) and cord inflammation (increased IL1β, IL8, IL4, IL10, MCP, MIP1α). The study was terminated because three patients developed delayed wound infections. At 18-months, two patients improved and three stayed the same. We conclude that, after TSCI, hypothermia is potentially beneficial by reducing cord inflammation, though after rewarming these benefits are lost due to increases in cord swelling, ischemia and inflammation. We thus urge caution when using hypothermia–rewarming therapeutically in TSCI.

## Introduction

Hypothermia is being investigated as a potential therapy for traumatic brain injury (TBI)^1^ and traumatic spinal cord injury (TSCI)^2^ based on compelling evidence from several laboratories and animal species that hypothermia is neuroprotective. In animal models of TBI, hypothermia has been shown to target many pathological processes including reducing the metabolic rate, inflammation, edema, oxidative stress, excitotoxicity, electrolyte imbalance as well as apoptotic and necrotic cell death in vulnerable tissue^3–8^. Despite the encouraging findings of animal studies, randomized controlled human trials have failed to show functional benefit of hypothermia in TBI patients^1,9,10^. Therapeutic hypothermia is also beneficial in animal models of TSCI: rodent models of TSCI show improved histological and functional outcomes after hypothermia^11,12^. Small, non-randomized studies of TSCI patients also suggest improved functional outcomes after local^13,14^ or systemic hypothermia^2,15^. To date, there are no published randomized controlled trials of hypothermia for TSCI.

Despite the wide interest in therapeutic hypothermia for CNS injuries, there is paucity of mechanistic data from humans regarding the effect of hypothermia on the injury site. In TSCI patients, the effect of cooling and rewarming on cord swelling, metabolism and inflammation are unknown. It is thus unclear if hypothermia and rewarming have beneficial or adverse effects on the injured human spinal cord. The lack of mechanistic information from TSCI patients is due to the lack of advanced monitoring from the injury site. Our group has developed techniques to monitor intraspinal pressure (ISP), spinal cord perfusion pressure (SCPP) and tissue metabolism from the injury site^16–23^. Here, we applied our monitoring techniques to define the effect of local hypothermia and rewarming on injury site physiology, metabolism and inflammation in TSCI patients.

## Methods

### Institutional research board approvals

The procedures followed were in accordance with the ethical standards of the responsible committee on human experimentation (institutional and national) and with the Helsinki Declaration of 1975, as revised in 2000 (World Medical Association Declaration of Helsinki 2000). Hypothermia–rewarming was approved as a sub-study (Amendment 7) of the ISCoPE (Injured Spinal Cord Pressure Evaluation) study for TSCI. Written consent was obtained from all patients. The hypothermia study, including the patient information sheet and the consent form, was approved by the St George’s, University of London Joint Research Office and the National Research Ethics Service London–St Giles committee (no. 10/H0807/23). ISCoPE is registered as NCT02721615 at UK Clinical trials gateway since 01/09/2010 and at ClinicalTrials.gov since 29/03/2016. The study protocol, patient consent form, patient information sheet and Amendment 7 are enclosed.

### Inclusion/exclusion criteria

This prospective patient cohort was recruited between September 2016 and February 2017. Inclusion criteria were: (1) Severe TSCI defined as American spinal injuries association Impairment Scale (AIS) grade A, B, or C; (2) Age 18–70 years; (3) Surgery within 72 hours of TSCI; (4) Thoracic injury (we excluded cervical injuries because of the length of the cooling balloon). Exclusion criteria were: (1) Other major co-morbidities or concurrent injuries; (2) Penetrating TSCI; (3) No consent.

### Clinical assessments and investigations

Examinations were performed according to the International Standards for Neurological Classification of Spinal Cord Injury (ISNCSCI) on admission, before discharge to the spinal rehabilitation facility (4–8 weeks) and during outpatient follow-ups (6–29 months). The patients were assessed by a neurosurgical TSCI research fellow trained in the ISNCSCI examination. CT and MRI scans of the whole spine were obtained on admission, a post-operative CT was done at 24–48 hours after surgery and a post-operative MRI after probe removal. On the Neuro-Intensive Care Unit (NICU), core temperature was recorded continuously from a urinary catheter temperature probe.

### Spinal fixation

Surgery was done posteriorly and aimed to restore the normal spinal alignment with laminectomies for bony decompression. Pedicle screws were inserted two levels above and two levels below the fracture and were linked with rods secured with blockers, using the Xia 3 system (Stryker, Newbury, UK).

### ISP probe and microdialysis catheter

An ISP probe (Codman Microsensor Transducer, Depuy Synthes, Leeds, UK) and a MD catheter (CMA 61, CMA microdialysis AB, Sweden) were tunnelled through the skin into the wound cavity. Under the operating microscope, the dura and arachnoid were opened one level below the injury. The ISP probe and MD catheter were inserted through the durotomy and advanced on the cord surface until the tips were at the site of maximal cord swelling. The dural opening was sutured and supplemented with Tisseel (Baxter, UK). The ISP probe and MD catheter were sutured to the skin. These techniques are described in detail in our earlier publications^16–23^.

### Cooling catheter

A sterile cooling balloon, part of a closed catheter loop system (Cool line catheter, Zoll medical, Cheshire, UK), and sterile thermometer (Smiths Medical, Ashford, UK) were tunnelled into the wound cavity and placed extradurally. A wound drain was inserted for a week and was set ‘under gravity’. The wound was closed in layers using vicryl sutures with nylon sutures for the skin. We gave 5,000 units dalteparin daily, starting at 24 hours after surgery. Dalteparin was omitted at 24 hours before and at 6 hours after removing the probes and wound drain. Prophylactic intravenous vancomycin/gentamicin were administered for 48 hours as per hospital protocol. In NICU, the wound was covered with an electric warming blanket set at 37 °C (CosyTherm mattress, Inspiration healthcare, Rotherham, UK).

### Monitoring setup

The ISP probe was connected to a Codman ICP box linked by a ML221 amplifier to a PowerLab running LabChart software (version 7.3.5; ADInstruments). Arterial blood pressure was recorded from a radial artery catheter connected to the Philips Intellivue MX800 bedside monitoring system (Philips, Guildford, UK) in turn connected to the PowerLab system. ISP and arterial blood pressure signals were sampled at 1 kHz for 7 days. LabChart was used to analyze the signals and compute SCPP, defined as MAP minus ISP. We previously showed that ISP measured this way is different from intrathecal pressure measured above or below the injury site because the swollen, injured cord is compressed against the surrounding dura thus compartmentalising the intrathecal space^18,22,23^. MD monitoring was started postoperatively in the NICU. CNS perfusion fluid (CMA Microdialysis AB, Sweden) was perfused at 0.3 µL/min using the CMA106 infusion pump (CMA Microdialysis AB, Sweden). MD vials were changed hourly and stored at 4 °C. The first two samples from each patient were discarded to allow priming of the MD catheter and stabilization of metabolite concentrations. Samples were batch-analyzed up to 24 hours later using ISCUS Flex (CMA Microdialysis AB, Sweden) for glucose, lactate, pyruvate, glycerol, glutamate and lactate-to-pyruvate ratio (LPR). 100-fold changes in metabolite concentration, and 10-fold changes in LPR, compared with the preceding hour, were excluded. We previously showed that our MD method measures injury site surface metabolism, which correlates well with intraparenchymal metabolism (r = 0.56–0.91), but differs from metabolites measured from the lumbar cerebrospinal fluid (CSF) (r = 0.23–0.50)^16–18^.

### Cytokines/chemokines

We measured the concentrations of cytokines/chemokines in the MD fluid collected from the injury site. These molecules were selected because they have been implicated in the pathophysiology of spinal cord injury (see supplement), they are <30 kDa (i.e. can cross the MD membrane) and they are part of our multiplex electrochemiluminescence assay panel. For each patient, MD vials were grouped by sampling period: 6 hours pre-cooling, 6 hours at 33 °C and 6 hours post-rewarming i.e. at 37 °C. MD vials, each containing about 10 μL, were stored at −80 °C and analyzed in a blinded fashion at Charité-Universitätsmedizin Berlin by multiplex electrochemiluminescence assays (Meso Scale Discovery, Rockville, MD, USA) using the following antibody sets from the U-PLEX Biomarker Group 1 Human Assays panel (K15067L): IL1α, IL1β, IL4, IL8, IL10, IP10, GROα, MCP1, MIP1α, MIP1β. If necessary, the MD samples were diluted to the minimum required volume for a single determination (25 µl) or analyzed directly according to the manufacturer’s protocol. 96-well plates including samples, blanks and recombinant standard concentrations were measured and unknown concentrations calculated, using the MESO QuickPlex SQ 120 Reader and the MSD DISCOVERY WORKBENCH software version 4.0 (Meso Scale Discovery, Rockville, MD, USA), respectively.

### Cooling-rewarming

A closed temperature system pumping sterile saline into the balloon catheter (Thermoguard XP temperature management system, Zoll Medical, Cheshire, UK) was connected at the bedside in NICU. Cooling was started at 12 hours after surgery, thus allowing baseline cord physiological and MD measurements to be obtained. In the first patient, the target temperature was reduced by 1 °C every 12 hours (37 to 33 °C). This slow reduction allowed real-time analysis of microdialysis samples and repeat neurological examinations to ensure no deterioration. After 12 hours at 33 °C, the cord was rewarmed to 37 °C at 0.5 °C/hour. In subsequent patients, the cord was cooled to 33 °C in one step and, after 72 hours, rewarmed at 0.5 °C/hour to 37 °C.

### Data analysis and statistics

For ISP, SCPP and metabolites, we compared the 12 values at 37 °C immediately preceding hypothermia (taken hourly) *vs*. the 12 values at 33 °C (taken hourly) *vs*. the first 12 values at 37 °C i.e. post-rewarming (taken hourly). For cytokines/chemokines, we compared the 6 values at 37 °C immediately preceding hypothermia (taken hourly) *vs*. 6 values at 33 °C (taken hourly) *vs*. the first 6 values at 37 °C i.e. post-rewarming (taken hourly). In each case, the three temperature groups (pre-hypothermia, hypothermia, post-rewarming) were compared pairwise by Kruskal-Wallis + post-hoc Dunn-Bonferroni. Analysis was done using XLStat Biomed version 2018.1 for Mac (Addinsoft, Paris, France). Statistical significance was taken as *P* < 0.05.

## Results

### Patient details

**Figure 1:**
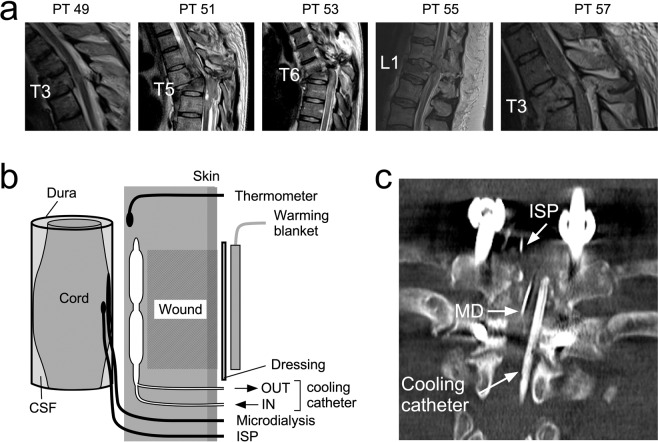
Setup. (**a**) Sagittal T2 pre-operative spinal MRI of the five patients (ISCoPE No. 49, 51, 53, 55, 57). Label shows fractured vertebra. (**b**) Schematic of setup for monitoring and cooling produced using Canvas Draw 3 for MacOS, https://www.canvasgfx.com/en/products/canvas-draw/. ISP probe and microdialysis catheter lie intradurally, on the surface of the injured cord. Cooling catheter and thermometer are extradural. A heating pad is placed on the skin. (**c**) Post-operative spinal CT showing ISP probe, microdialysis (MD) and cooling catheters.

We recruited five consecutive patients with severe thoracic TSCIs (Fig. 1). Most were young (aged 45.4 ± 7.1 years, mean ± standard error) and male (66.7%). The delay between TSCI and surgery was 47.4 ± 9.6 hours and the injury to cooling time 70.4 ± 9.3 hours. At 18.2 ± 5.1 months follow up, total motor score increased by 8.8 ± 8.5, total pin prick score by 6.8 ± 6.5 and total light touch score by 2.6 ± 2.5 points. Overall, two patients (40%) had improved by at least one AIS grade. For further details see Table 1 and Fig. 1a. Subsequent analysis includes all patients.

**Table 1:** Demographics of the five patients in the study.

PT	Age	Sex	Injury to surgery (hours)	Injury to cooling (hours)	Injury level	Admission				Followup				Followup (months)
						AIS	TMS	TPP	TLT	AIS	TMS	TPP	TLT	
49	66	F	65	88	T3	B	50	54	54	D	89	83	61	29
51	36	M	49	71	T5	A	50	52	52	A	50	48	48	25
53	30	M	21	45	T6	A	50	52	52	A	50	52	52	22
*55	52	F	36	60	L1	A	51	81	80	B	56	88	88	6
57	43	M	66	88	T3	A	50	40	40	A	50	42	42	9

F, Female; M, Male; AIS, American spinal injuries association Impairment Scale; PT, patient; TLT, total light touch score; TMS, total motor score; TPP, total pin prick score; *No cooling, equipment failure.

### Cooling–rewarming

**Figure 2:**
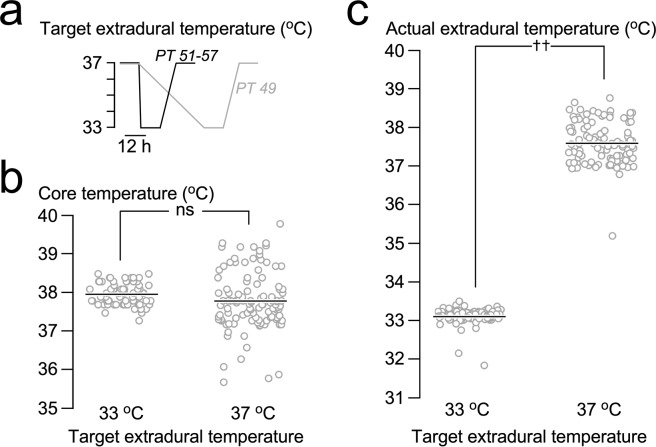
Relations between various temperatures. (**a**) Extradural cool/rewarm regime. (**b**) Core temperature at target extradural temperatures 33 and 37 °C. (**c**) Actual extradural temperature at target extradural temperatures 33 and 37 °C. (**b,c**) Show raw data and mean. Not significant ns, *P* < 0.0001^††^.

Local hypothermia and rewarming were well tolerated without causing pain, discomfort or shivering. Throughout the cooling and rewarming phases, all patients remained alert, orientated and self-ventilating with no changes in their neurological examinations. Details of our setup for cooling–rewarming and monitoring are shown in Fig. 1b,c. Except for the first patient (PT 49), who was subjected to a slow cooling protocol for safety, all subsequent patients reached the target extradural temperature of 33 °C in 20 minutes. In all patients, the cooling protocol reduced the extradural temperature to the target value without causing a significant change in the core temperature (Fig. 2).

### Injury site physiology

**Figure 3:**
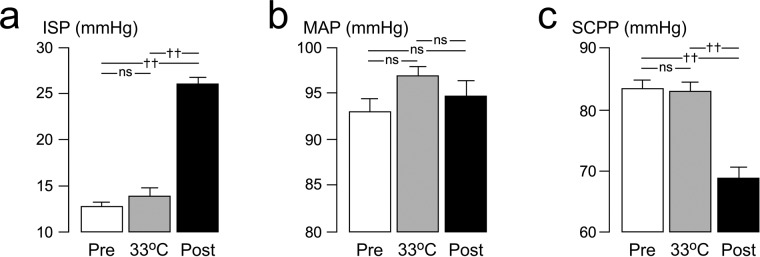
Effect of cooling-rewarming on ISP, MAP and SCPP. (**a**) ISP, (**b**) MAP and (**c**) SCPP for 12 h before start of cooling (white, Pre), for 12 h at minimum temperature (gray, 33 °C) and for 24 h after the end of rewarming (black, Post). Mean + /− standard error. Not significant, ns; *P* < 0.0001^††^.

In the 12 hours before the start of the cool–rewarm protocol, baseline spinal cord ISP was 12.7 mmHg and SCPP at 83.3 mmHg with MAP at 93.1 mmHg. Cooling from 37 to 33 °C did not produce any significant changes in ISP, SCPP or MAP. Rewarming from 33 to 37 °C was associated with significant increase in ISP by 13.2 mmHg and significant decrease in SCPP by 14.6 mmHg compared with corresponding average pre-cooling baseline levels, whereas MAP remained unaffected. For further details see Fig. 3.

### Injury site metabolism

**Figure 4:**
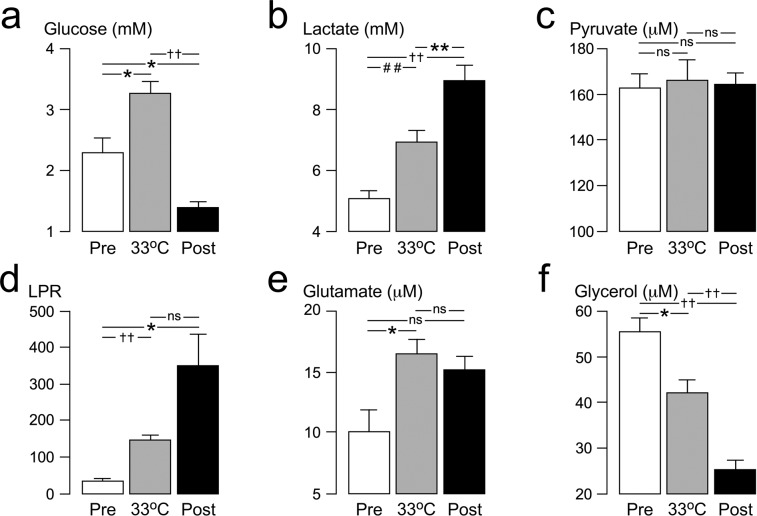
Effect of cooling-rewarming on injury site metabolites. (**a**) Glucose, (**b**) Lactate, (**c**) Pyruvate, (**d**) Lactate-to-pyruvate ratio (LPR), (**e**) Glutamate and (**f**). Glycerol. Metabolite levels for 12 h before start of cooling (white, Pre), for 12 h at minimum temperature (gray, 33 °C) and for 24 h after the end of rewarming (black, Post). Mean + /− standard error. ns not significant, *P* < 0.05^*^, <0.001^##^, <0.0001^††^.

In the 12 hours before the start of the cool-rewarm protocol, baseline spinal cord tissue glucose concentration was 2.3 mM, tissue lactate 5.1 mM, tissue pyruvate 148.5 μM, tissue LPR 33.9, tissue glutamate 10.9 μM and tissue glycerol 54.5 μΜ. Cooling and rewarming had a major impact on injury site metabolism. Cooling was associated with significant increase in tissue glucose by 1.0 mM, significant increase in lactate by 1.9 mM, no change in pyruvate, significant increase in LPR by 113.0, significant increase in glutamate by 6.4 μM and significant decrease in glycerol by 13.4 μM. Compared with average pre-cooling baseline levels, rewarming was associated with significant decrease in tissue glucose by 0.9 mM, significant increase in lactate by 3.9 mM, no change in pyruvate, significant increase in LPR by 314.9, no change in glutamate by 6.4 μM and significant decrease in glycerol by 30.0 μM. For details see Fig. 4.

### Injury site inflammation

**Figure 5:**
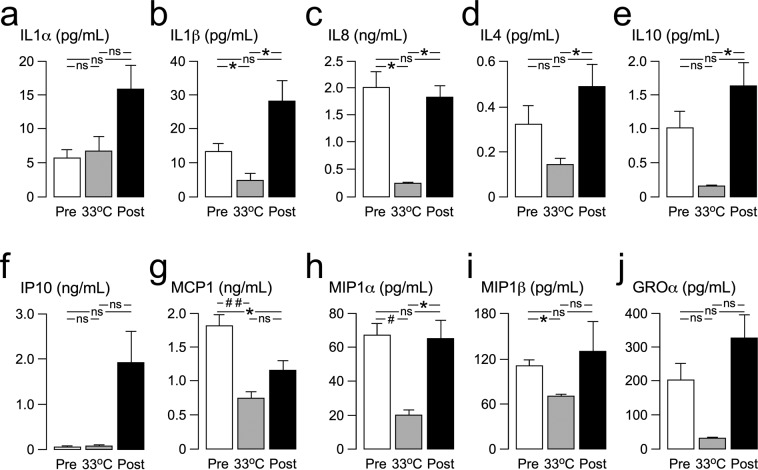
Effect of cooling-rewarming on injury site inflammatory mediators. (**a**) IL1α, (**b**) IL1β, (**c**) IL8, (**d**) IL4, (**e**) IL10, (**f**) IP10, (**g**) MCP1, (**h**) MIP1α, (**i**) MIP1β and (**j**). GROα. Mediator levels for 12 h before start of cooling (white, Pre), for 12 h at minimum temperature (gray, 33 °C) and for 24 h after the end of rewarming (black, Post). Mean + /- standard error. Not significant ns, *P* < 0.05^*^, <0.005^#^, <0.001^##^.

In the 12 hours before the start of the cool-rewarm protocol, baseline spinal cord IL1α concentration was 5.6 pg/mL, tissue IL1β was 13.5 pg/mL, tissue IL8 was 2,013.9 pg/mL, tissue IL4 was 0.3 pg/mL, tissue IL10 was 1.0 pg/mL, tissue IP10 was 66.4 pg/mL, tissue MCP1 was 1,821.5 pg/mL, tissue MIP1α was 67.0 pg/mL, tissue MIP1β was 111.2 pg/mL and tissue GROα was 268.8 pg/mL. The concentrations of injury site inflammatory cytokines significantly decreased with cooling by 62.8% for IL1β, 87.1% for IL8, 58.7% for MCP1, 69.8% for MIP1α and 37.0% for MIP1β. Compared with hypothermia, there was rebound significant increase in the concentrations of inflammatory cytokines on rewarming by 465 .3% for IL1β, 606.1% for IL8, 241.6% for IL4, 848.1% for IL10 and 222.4 for MIP1α. A markedly, though statistically non-significant rise in cytokine/chemokine concentration after rewarming was also observed for IL1α, IP10, and GROα. Compared with baseline, after rewarming the levels of IL1α, IL1β, IL4, IL10, IP10, MIP1β and GROα were higher whereas the levels of IL8, MCP1 and MIP1α were lower; these changes were not significant except for MCP1. For details see Fig. 5.

### Complications

Spinal cord cooling and rewarming was associated with complications, summarized in Table 2. In 1/5 (20%) patients, the equipment failed; there was a hole in the cooling catheter balloon that allowed the cooled sterile saline fluid to enter the wound cavity and leave through the wound drain. In this patient, cooling was stopped and the catheter was removed. Three of the five (60%) patients had culture positive delayed wound infections; in two of the three patients, we performed wound washout under general anesthetic. These three patients received antibiotics with the metalwork *in situ* and recovered with no deterioration in ISNCSCI neurological scores. In contrast, we have reported no wound infections in 42 consecutive patients that had spinal cord monitoring without hypothermia^21^. After discussion with the sponsors was well as our neurosurgical, intensive care unit and microbiology colleagues, the decision was taken to prematurely terminate the study.

**Table 2:** Wound complications of localized hypothermia.

Patient	Wound breakdown	Wound bacteria	Surgical wound treatment	Antibiotics	Outcome
49	Yes	*E. coli S. aureus*	Day 16 lumbar drain Day 43 debridement	52 days	Wound well-healed
51	No	No	No	No	Wound well-healed
53	No	*E. coli*	No	45 days	Wound well-healed
*55	No	No	No	No	Wound well-healed
57	Yes	*S. aureus*	Day 22 pus aspirated Day 24 debridement	56 days	Wound well-healed

*No cooling, equipment failure.

### Outcomes

At a mean follow-up of 36 weeks, 2/5 (40%) patients improved AIS grade, one from A to B and the other from B to D. We observed increase in mean ISNCSCI total motor score by 8.8 points (0–39), pin prick sensation of 6.8 points (−4–29) and total light touch sensation of 2.6 points (−4–8). The wounds appeared well-healed at follow-up.

## Discussion

We employed local hypothermia aiming to avoid the side effects of systemic hypothermia i.e. coagulopathy, electrolyte disturbances, myocardiac ischemia, atrial fibrillation, sepsis, pneumonia and altered drug metabolism^24^. Our key findings are that, during hypothermia–rewarming in awake TSCI patients, there is no discomfort or shivering and no change in ISNCSCI motor and sensory scores. The hypothermia phase was potentially beneficial, by reducing injury site inflammation, though during the rewarming phase these benefits were lost due to rebound increases in injury site swelling, ischemia and inflammation. The study was terminated early because of delayed wound infections in 60% of patients. The major weaknesses of our study are the small sample size and the lack of a control group.

In a previous study of local hypothermia, cooling was induced within 7 hours of TSCI at a rapid rate using an extradural device set at 6 °C; hypothermia lasted 3–4 hours with rapid rewarming^13^. In comparison, we started hypothermia later on, cooled within 20 min (apart from the first patient) to 33 °C for 12 hours and rewarmed slowly at 0.5 °C/h. It has been suggested that slower rewarming (<0.5 °C/h) might be more beneficial^25^. We do not know whether earlier onset hypothermia or more prolonged hypothermia or slower rewarming might have shown beneficial effects on ISP, SCPP, cord metabolism and cord inflammation. Because our study was terminated early; we were unable to study the effects of these changes on the injured spinal cord.

Hypothermia had no significant impact on baseline ISP, whereas rewarming was associated with a marked rebound rise in ISP by 13.2 mmHg. Since the MAP remained constant throughout, the ISP rise caused a marked drop in SCPP by 14.6 mmHg. These findings mirror observations in human TBI, where hypothermia caused non-significant decrease in intracranial pressure (ICP)^9,10^ and rewarming caused rebound rise in ICP^26^. The effects of hypothermia–rewarming on ICP may be related to blood flow changes, i.e. vasoconstriction during hypothermia and vasodilatation during rewarming^27^. In our patients, baseline ISP was not elevated, at 12.7 mmHg, which may explain why hypothermia did not reduce ISP. The ISP rise we observed after rewarming may have been caused by increased inflammation within the injured cord related to reperfusion. There is substantial evidence that during reperfusion, leukocytes become activated to release multiple inflammatory factors^28^, consistent with the marked rise in the tissue cytokines/chemokine levels observed here.

Hypothermia was associated with a rise in tissue glucose, lactate and LPR without change in pyruvate. Rewarming was associated with a marked fall in tissue glucose, rise in lactate and LPR, again with constant pyruvate. Hypothermia is known to reduce cellular metabolism and, in brain, for every 1 °C drop in temperature, there is a 6% drop in cerebral metabolic rate, which is expected to reduce glucose consumption at the injury site^27^, thus explaining the rise in tissue glucose concentration. After rewarming, the cord tissue glucose level fell probably due to increased cellular utilisation. In the brain, lactate is not only a by-product of anaerobic metabolism, but may also be a neuronal energy substrate^29^. Several mechanisms may thus account for the rise in tissue lactate, without change in tissue pyruvate, during hypothermia and rewarming e.g. depleted O_2_ supply thus increased anaerobic metabolism, failure of pyruvate dehydrogenase thus diverting pyruvate to lactate rather than into the tricarboxylic acid cycle and increased uptake of lactate at the injury site from the circulation. The relative contributions of these mechanisms may differ in hypothermia *vs*. rewarming. Whatever the underlying mechanisms, the main finding here is that hypothermia–rewarming is associated with a marked impairment of metabolism at the injury site.

Hypothermia–rewarming was also associated with rise in tissue glutamate and fall in tissue glycerol. Glutamate is an excitatory amino acid that causes excitotoxic damage to neurons, whereas glycerol is released into the extracellular fluid during plasma cell membrane lysis^30^. Interestingly, during the 12 hours before the onset of hypothermia, tissue glutamate was rising at a rate of 1.3 μM/h, whereas glycerol was decreasing at a rate of 2.6 μM/h. One possibility, therefore, is that changes in tissue glutamate and glycerol largely represent trends related to the timing from the injury rather than the effects of hypothermia or rewarming. In human TBI, hypothermia–rewarming is generally associated with reduced ventricular CSF glutamate^31^. Other possibilities to explain our observation of increased MD glutamate during hypothermia in TSCI are lack of strong correlation between CSF *vs*. MD glutamate, as we have recently shown^32^, or even differences in the cell-level responses to traumatic injury of the spinal cord compared with the brain^33^.

A striking finding in our study is the marked reduction in the concentrations of tissue cytokines/chemokines during hypothermia followed by rebound enhancement of tissue inflammation associated with rewarming, generally to above pre-hypothermia baselines. Several cell types likely secrete cytokines at the injury site including neutrophils, macrophages, microglia, lymphocytes, astrocytes and endothelial cells. The differential responses of these cytokines to hypothermia-rewarming may be due to differential responses of these cell types to hypothermia-rewarming. The effect of hypothermia in suppressing inflammation after TSCI mirrors findings in animal models^34^ though the effect of rewarming on inflammation has not been investigated. On the one hand, the suppression of inflammation induced by hypothermia may be beneficial to the damaged spinal cord, but on the other hand it may predispose to wound infection. An earlier study reported a wound infection rate of 10%^13^ and our study found 60% wound infection rate. Potential ways to reduce wound infection risk in future trials include prolonged antibiotics and iodine impregnated occlusive dressings. The rebound increase in tissue inflammation is potentially detrimental to the injured cord e.g. by increasing vasogenic edema from breakdown of the blood-spinal cord barrier^28^.

Based on our findings here, we urge caution when performing trials of hypothermia for TSCI without simultaneously monitoring from the injury site to determine in real time the impact on the injured spinal cord.

## Supplementary information

Supplementary Information.

## Data Availability

All data generated or analyzed during this study are included in this published article.
