# Supplementary material for: Effects of local hypothermia–rewarming on physiology, metabolism and inflammation of acutely injured human spinal cord
Source: Sci Rep. 2020 May 15;10:8125. doi: 10.1038/s41598-020-64944-y (PMC7229228; doi:10.1038/s41598-020-64944-y)
Supplement: Supplementary file 1 — Supplementary Information. [file 41598_2020_64944_MOESM1_ESM.pdf]

Effects of local hypothermia–rewarming on physiology, metabolism and inflammation of acutely injured human spinal cord

Mathew J. Gallagher, Florence R.A. Hogg, Siobhan Kearney, Marcel Kopp, Christian Blex, Leonarda Serdani Oliver Sherwood, Jan M. Schwab, Argyro Zoumprouli, Marios C. Papadopoulos, Samira Saadoun

SUPPLEMENT

CYTOKINE	PRO or ANTI INFLAMMATORY	SECRETED BY	EFFECTS	ROLE IN SCI	CV %
**IL1α**	PRO	Macrophages, Neutrophils, Epithelial cells, Endothelial cells	Production of inflammation<br>Fever<br>Sepsis	*J Neurosci.* 2015;35:10715-30. IL1α deletion protects oligodendrocytes after SCI. *J Neuroinflammation.* 2012;9:65. IL1α activates microglia/macrophages after SCI	16.5
**IL1β**	PRO	Macrophages, Neutrophils, Epithelial cells, Endothelial cells	Production of inflammation<br>Fever<br>Sepsis	*Spine.* 2004;29:966-71. IL1β is upregulated in human SCI. *PNAS.* 2014;111:8263-8. IL1β may cause chronic pain after SCI	3.6
**IL4**	ANTI	T cells<br>Basophils<br>Mast cells	Regulates humoral + adaptive immunity. Decreases production of Th1 cells, macrophages, IFNγ and IL12. Promotes activation of macrophages into M2 cells and inhibits activation into M1 cells	*J Neurosci Res.* 2010;88:2409-19. IL4 reduces cavity formation after SCI. *Pharmaceuticals.* 2017;10(4). Systemic IL4 administration is neuroprotective after SCI	7.3
**IL8**	PRO	Macrophages, Neutrophils, Endothelial cells	Induces chemotaxis in neutrophils and other granulocytes, causing them to migrate toward the site of infection and stimulates phagocytosis once they have arrived	*J Neurotrauma.* 2010;27:669-82. IL8 is upregulated in human SCI	2.2
**IL10**	ANTI	Macrophages<br>Lymphocytes	Suppresses cytokine secretion, antigen presentation and CD4+ T cell activation and inhibits lipopolysaccharide and bacterial mediated induction of pro-inflammatory cytokines	*J Neuroinflammation.* 2019;16:93. IL10 limits inflammation & improves outcome after SCI. *J Neurotrauma.* 2013;30:1311-24. IL10 is beneficial after SCI	15.1
**IP10**	PRO	Macrophages, Endothelial cells Fibroblasts	Secreted in response to IFNγ. Promotes chemoattraction for macrophages, T cells, NK cells and dendritic cells and promotes T cell adhesion to endothelial cells	*J Neurosci Res.* 2006;84:724-34. Neutralization of IP10 reduces apoptosis and increases axon sprouting after SCI. *J Neurosci Res.* 2004;77:701-8. Neutralization of IP10 enhances tissue sparing and angiogenesis following SCI	2.7
**GROα**	PRO	Macrophages, Neutrophils, Epithelial cells	Neutrophil chemoattraction	*J Cell Physiol.* 2012;227:1335-46. Increased expression after SCI. *J Neurochem.* 2001;78:1064-72. Neurtrophil chemoattraction after SCI and role of GROα	5.5
**MCP1**	PRO	Macrophages<br>Dendritic cells	Recruits macrophages, memory T cells and dendritic cells to the sites of inflammation produced by tissue injury or infection	*J Neurotrauma.* 2010;27:669-82. MCP1 is upregulated in human SCI. *J Neurosci Res.* 2002;68:691-702. Monocyte recruitment + myelin removal delayed after SCI in mice with non-functional MCP1	5.4
**MIP1α**	PRO	Macrophages	Recruitment and activation of polymorphonuclear leukocytes	*J Neurosurg Spine.* 2011;14(5):583-97. Increased MIP1α expression after SCI. *J Neurosurg Spine.* 2011;14(5):583-97. Increased MIP1α expression after SCI	14.3
**MIP1β**	PRO	Macrophages	Chemoattractant for natural killer cells, monocytes and a variety of other immune cells.	*J Neurosci.* 2017;37(48):11731-11743. Increased MIP1β after SCI. *Spinal Cord.* 2017;55(11):1002-1009. Increased MIP1β after SCI	4.5

CV = Coefficient of Variation of assay.
